# Improved Prediction of Hourly PM_2.5_ Concentrations with a Long Short-Term Memory Optimized by Stacking Ensemble Learning and Ant Colony Optimization

**DOI:** 10.3390/toxics13050327

**Published:** 2025-04-23

**Authors:** Zuhan Liu, Xianping Hong

**Affiliations:** 1 School of Information Engineering, Nanchang Institute of Technology, Nanchang 330099, China; xianping1217@foxmail.com; 2 Jiangxi Province Key Laboratory of Smart Water Conservancy, Nanchang 330099, China

**Keywords:** PM_2.5_, long short-term memory network (LSTM), ant colony optimization (ACO), stacking ensemble learning, deep learning

## Abstract

To address the performance degradation in existing PM_2.5_ prediction models caused by excessive complexity, poor spatiotemporal efficiency, and suboptimal parameter optimization, we employ stacking ensemble learning for feature weighting analysis and integrate the ant colony optimization (ACO) algorithm for model parameter optimization. Combining meteorological and collaborative pollutant data, a model (namely the stacking-ACO-LSTM model) with a much shorter consuming time than that of only long short-term memory (LSTM) networks suitable for PM_2.5_ concentration prediction is established. It can effectively filter out feature variables with higher weights, thereby reducing the predictive power of the model. The prediction of hourly PM_2.5_ concentration of the model is trained and tested using real-time monitoring data in Nanchang City from 2017 to 2019. The results show that the established stacking-ACO-LSTM model has high accuracy in predicting PM_2.5_ concentration, and compared to the same model without considering time and space efficiency and defective parameter optimization, the mean square error (MSE) decreases by about 99.88%, and the coefficient of determination (R^2^) increases by about 2.39%. This study provides a new idea for predicting PM_2.5_ concentration in cities.

## 1. Introduction

In recent years, the air quality has deteriorated and the phenomenon of haze has increased, posing a greater threat to human health. The main factor of air pollution is inhalable particulate matter, led by PM_2.5_. Epidemiological studies demonstrate that prolonged exposure to elevated PM_2.5_ levels significantly increases risks of pulmonary diseases, cardiovascular disorders, and malignancies, while also influencing radiative forcing and climatic patterns [1,2]. Precise PM_2.5_ forecasting enables proactive public health protection and supports evidence-based environmental policy formulation.

The algorithms of PM_2.5_ concentration prediction are mainly divided into two categories: deterministic and artificial intelligence models. Deterministic models, including WRF [3,4] and CMAQ [5,6], simulate atmospheric processes to predict pollutant concentrations through physical and chemical mechanisms [7,8]. However, deterministic models require good computing resources and a meteorological knowledge background, and there are the following shortcomings. In terms of input PM_2.5_ samples, their stability is insufficient, which means that the quality and reliability of the data are difficult to guarantee and may introduce a large amount of noise and errors. Excessive computational demands significantly compromise prediction efficiency and often preclude real-time application. Furthermore, these models exhibit unstable performance with substantial scenario-dependent variability. Excessive computational demands significantly compromise prediction efficiency and often preclude real-time application. Furthermore, these models exhibit unstable performance with substantial scenario-dependent variability. The lack of accuracy also makes it difficult for the prediction results to effectively guide decision-making and planning. These issues seriously constrain the application and development of traditional prediction methods in the field of PM_2.5_ concentration prediction. By comparison, artificial intelligence models based on deep learning perform well in air quality prediction, such as support vector machines (SVM) [9], BP neural networks [10], extreme learning machines (ELM) [11,12], and so on. But these feedforward models are not adjusted according to the specific situation of the dataset and are more suitable for time-series and real-time updating of data. The LSTM are gradually entering the field of scholars.

LSTM, as an important architecture in deep learning, displays unique advantages in dealing with time-series data. The reason is that LSTM can effectively capture the long-term dependencies in time-series data, which is very crucial for PM_2.5_ concentration data that varies over time. By using the obtained air quality data to train the LSTM model, the model can learn the inherent laws and trends in the data. The LSTM-based approach utilizes PM_2.5_ data along with ancillary data, meteorological variables from the Copernicus Atmospheric Monitoring Service operated by ECMWF, and temporal variables related to local emissions to improve air pollution forecasting performance. Bedi [13] and Bedi et al. [14] utilized recurrent neural networks with long short-term memory (LSTM) units to predict Delhi’s PM_2.5_ concentration and showed that the model combining PM_2.5_ data with gaseous pollutants as well as the model combining PM_2.5_ data with gaseous pollutants and meteorology predicted daily and hourly PM_2.5_ concentration more accurately than other models. However, LSTM models are highly dependent on the inputs predicted by the numerical model, which reflects a common problem of using a single model for prediction, namely the low prediction accuracy and long training time of the model. To address these issues, the concept of ensemble models has begun to be widely accepted. These novel ensemble forecast models overcome these drawbacks by simultaneously taking into account both forecast accuracy and stability [15]. For example, applying only the LSTM model to the CMAQ forecasts can yield reasonable forecast skill levels comparable to the operational AirKorea forecasts that elaborately combine the CMAQ model, AI models, and human forecasters [16]. Lin et al. [17] applied an application strategy, an innovative application-strategy-based LSTM (namely ASLSTM), on the BLSTM to customize ASLSTM for the short-term and accurate prediction of PM_2.5_ concentrations. Combining spatial weighting, empirical mode decomposition (EMD), and a long short-term memory (LSTM) network, Yu et al. [18] proposed an ensemble model to predict PM_2.5_ concentration, namely CBAM-CNN-Bi LSTM. By comparing with RNN, HPO-RNN, GRU, and LSTM solely, the results show that the new model surpasses five benchmark models in terms of prediction accuracy.

In addition, LSTM can be combined with other ensemble learning techniques for prediction, such as convolutional neural networks (CNNs) [19,20,21,22,23], graph convolution neural networks (GCNNs) [15,24], ensemble empirical mode decomposition (EEMD) [25,26], attention mechanism [26,27,28], osprey optimization algorithm (OOA) [29,30], hybrid integration (HIG) algorithm [31,32], temporal convolutional network (TCN) [27,33,34,35], transformer [15,36,37], graph sample and aggregation network (GraphSAGE) [15,38,39], bidirectional recurrent gated neural network (BiGRU) [22,40,41], adaptive boosting (AdaBoost) [42,43], genetic algorithm (GA) [44,45,46], *k*-nearest neighbors (*k*NN) [46,47,48], random forest (RF) [20,46], support vector regression (SVR) [46,49], and particle swarm optimization (PSO) [46,50,51].

In this study, we initially designed a basic LSTM-based prediction model for preliminary forecasting, selecting all monitoring stations in Nanchang as the baseline. As a representative urban area, Nanchang’s air quality data can reflect the impact of various environmental factors and human activities on PM_2.5_ concentrations. This model was used to predict PM_2.5_ levels in Nanchang over the coming days, providing a foundation for subsequent optimization efforts. The original LSTM model consumed significant computational resources during prediction and relied solely on a rolling forecast mechanism based on time steps, without extensive hyper parameter optimization. Given these limitations, we prioritized two key aspects when selecting optimization algorithms: reducing computational demands and improving efficiency. While Bayesian optimization was initially considered for overall model tuning, its inefficiency in large-scale parameter searches and high computational requirements made it less suitable. After thorough evaluation, we adopted a stacking ensemble learning approach to analyze feature variable weights, thereby reducing computational load and enhancing prediction accuracy. Additionally, we introduced the ant colony optimization (ACO) algorithm to optimize the model’s hyper parameters, preventing it from settling into local optima. Building on these improvements, we developed an integrated stacking-ACO-LSTM model, which significantly reduced runtime compared to the standalone LSTM network, making it well suited for PM_2.5_ concentration prediction. The results demonstrate that this model not only performs well in spatiotemporal efficiency and parameter optimization for Nanchang’s PM_2.5_ prediction but also exhibits superior forecasting performance and stronger generalization capabilities. It holds substantial practical value and provides meaningful real-world reference significance.

## 2. Materials and Methods

### 2.1. Data Source

The study is based on the hourly pollutant index data of the PM_2.5_, PM_10_, SO_2_, NO_2_, CO, O_3_, and AQI in Nanchang City from 1 February 2017 to 30 September 2019, which are published on the China Air Quality Online Monitoring and Analysis Platform (https://www.aqistudy.cn). As for why Nanchang City was chosen as the research object, there are mainly the following reasons: Firstly, the data availability is high, and the channels for obtaining data of Nanchang City are very convenient. Secondly, compared with other prefecture-level cities, Nanchang City has more complete air quality monitoring stations, which can comprehensively and accurately collect various pollutant data. Thirdly, as the capital city of Jiangxi Province, Nanchang has a large population and active economic activities, and all these factors will have a significant impact on the air quality.

The relationships among the data: In nature, there are more than a dozen factors that affect PM_2.5_, such as wind speed, temperature, precipitation, industrial production, emissions from natural sources, coal combustion, and so on. However, in a certain area, some influencing factors tend to be constant within a fixed period of time. For example, in southern cities, the average monthly precipitation and wind speed do not vary much. Therefore, to some extent, factors like wind speed, temperature, and precipitation have a relatively small impact on the determination of PM_2.5_ content within a fixed area. As an industrial city, Nanchang’s industrial production process emits waste gases, which will lead to a continuous increase in the contents of PM_10_, CO, SO_2_, O_3_, and NO_2_ in the air. In view of this, in order to conduct analysis and prediction more efficiently, it is decided to select only these factors with more significant influences for in-depth study. In this way, we can focus our efforts on exploring the specific relationships and action mechanisms between them and PM_2.5_, thus providing more targeted evidence for a better understanding and control of the PM_2.5_ concentration.

Taking the data of PM_2.5_ concentration as an example, there should originally be a total of 23,304 pieces of PM_2.5_ concentration data, among which 1456 pieces of data are missing, accounting for approximately 6.25% of the total data. After removing the missing data, there are 21,848 pieces of data left. The length of the time series is 21,848, which is divided into training and test sets in a ratio of 8:2. For missing data, the commonly used filling methods usually include interpolation methods (such as linear interpolation, spline interpolation, etc.) or the average value method. They are filled in with the average value of nearby time points, which can ensure the integrity of the data and also make the filling and recharging conform to the characteristics of the overall data. Since the overall amount of missing data is not very large, and using the average value to fill in the missing data can also ensure the accuracy of the data to a large extent, using this method will basically not have an impact on the final experimental results. And due to the large scale of the data in the experiment, the data were standardized.

### 2.2. Long Short-Term Memory Network

**Figure 1:**
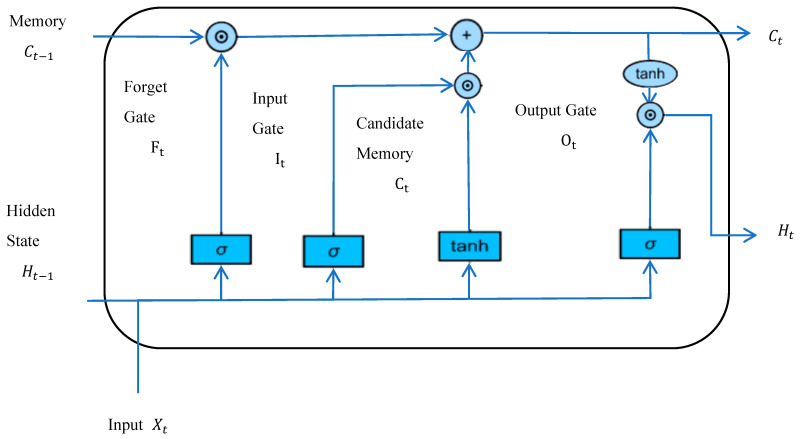
Basic framework of LSTM.

Long Short-Term Memory Network (LSTM) is an improved network framework based on recurrent neural network (RNN), which mainly solves the problem of insufficient processing of long-distance dependencies in RNN. The basic idea is based on the original RNN hidden layer having only one state sensitive to short-term inputs, plus a unit state that preserves long-term memory. LSTM achieves long-term state memory through the input gate, forget gate, and output gate. The gate structure selectively allows information to pass through, mainly through a sigmoid neuron and a pointwise multiplication operation. The first step in LSTM is to determine what information to discard from the cell state, which is accomplished through the forget gate. The next step is to determine how much new information to incorporate into the cellular state, which is accomplished through the input gate. Ultimately, it is necessary to determine what value to output, which is determined by the output gate. The basic framework of LSTM is shown in Figure 1.

In Figure 1, the shape of the storage unit (namely the vector dimension) is the same as the hidden state. It is designed to record additional hidden states and input information data. Some literature treats the storage cell as a special kind of hidden state. In particular, the input gate controls the addition of information from the input observation data at this moment and the hidden state from the previous moment to the storage cell. The forget gate controls what was forgotten in the storage cell at the previous moment. The output gate controls which information in the memory cell will be output to the hidden state.

**Figure 2:**
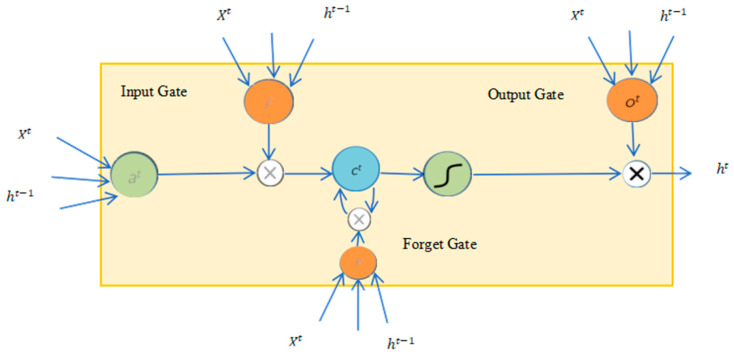
Forward propagation model.

To make it easier to understand the forward-propagation process of the LSTM model, we adapt the model structure diagram as shown in Figure 2, where $a^{t}$ refers to the candidate memory cell ${\tilde{C}}_{t}$ at time *t*.

From this, we can obtain the forward-propagation formula of the LSTM model:

(1) $$\left\{\begin{array}{c} \mathrm{Candidate memory cell}:\;\;\;{\tilde{C}}_{t}=\tanh\left(X_{t}W_{xc}+H_{t-1}W_{hc}+b_{c}\right),\;\;\;\;\;\;\;\;\;\;\;\;X_{t}\in R^{m\times d},H_{t-1}\in R^{m\times h},W_{xc}\in \;R^{d\times h}\\ \mathrm{Input Gate}:\;\;\;\;I_{t}=\sigma \left(X_{t}W_{xi}+,H_{t-1}W_{hi}+b_{i}\right),\;\;\;\;\;\;\;\;\;\;\;\;\;\;\;\;\;\;\;\;\;\;\;\;\;\;\;\;\;\;\;\;\;\;\;\;\;\;\;\;\;\;\;\;\;W_{xi}\in R^{d\times h},W_{hi}\in R^{h\times h}\\ \mathrm{Forget Gate}:\;\;\;\;F_{t}=\sigma \left(X_{t}W_{xf}+,H_{t-1}W_{hf}+b_{f}\right),\;\;\;\;\;\;\;\;\;\;\;\;\;\;\;\;\;\;\;\;\;\;\;\;\;\;\;\;\;\;\;\;\;\;\;\;\;\;\;\;\;\;\;W_{xf}\in R^{d\times h},W_{hf}\in R^{h\times h}\\ \mathrm{Output Gate}:{\;\;\;O}_{t}=\sigma \left(X_{t}W_{xo}+,H_{t-1}W_{ho}+b_{o}\right),\;\;\;\;\;\;\;\;\;\;\;\;\;\;\;\;\;\;\;\;\;\;\;\;\;\;\;\;\;\;\;\;\;\;\;\;\;\;\;\;\;\;W_{xo}\in R^{d\times h},W_{ho}\in R^{h\times h} \end{array}\;\;\right.$$

(2) $$\left\{\begin{array}{l} \;\;\;\;\;\;\;\;\mathrm{Memory cell}:{\;\;\;\;\;\;\;\;\;C}_{t}=I_{t}\;{\tilde{C}}_{t}+F_{t}\;\;C_{t-1}\;\\ \;\;\;\;\;\mathrm{Hidden state}:\;\;\;\;\;\;\;\;\;\;\;\;\;\;\;\;H_{t}=O_{t}\;\;\;\tanh(C_{t})\\ \;\;\;\mathrm{Model output}:\;\;{\hat{Y}}_{t}=H_{t}W_{hy}+b_{y},\;\;\;\;\;\;\;\;W_{hy}\in R^{h\times q},{\hat{Y}}_{t}\in R^{m\times q}\\ \mathrm{Loss function}:\;\;L=\frac{1}{T}\sum_{t=1}^{T}1\left({\hat{Y}}_{t},Y_{t}\right),\;\;\;\;\;\;\;\;L\in R \end{array}\right.$$

where *m* and *d* are the batch size of mini-batch stochastic gradient descent and the dimension of the word vector of the input word, respectively. At the same time, *h* and *q* are the vector widths (dimensions) of the hidden state and the model output.

### 2.3. Ant Colony Optimization

LSTM is prone to getting stuck in local optima and has a slow convergence speed [52,53]. Therefore, in order to optimize the hyperparameters of the PM_2.5_ concentration prediction model, the ant colony optimization (ACO) algorithm is introduced for parameter optimization, which is one of the most popular algorithms for hyperparameter optimization. The ACO is an essentially self-organizing, positive feedback parallel algorithm with strong robustness.

The ACO is a bionic algorithm derived from simulating the path-finding methods of natural ants [54,55]. Ants leave pheromones on the paths they pass through as they move, which are used to transmit information. In addition, ants can sense this pheromone substance during movement and use it to guide their direction of movement. Therefore, the collective behavior of an ant colony composed of a large number of ants shows a positive information feedback phenomenon. That is, the more ants have walked on a certain path, the greater the probability for later ants to choose this path. So, after a series of searches, the ant colony can finally find a logical shortest path.

At the initial moment of the algorithm, *m* ants are randomly placed in *n* cities. Meanwhile, the first element of each ant’s taboo list (*tabu_k_*) is set to the city where it is currently located. At this time, the amount of pheromone on each path is equal. Let $T_{ij}\left(0\right)$ = *c* (where c is a relatively small constant). At time *t*, the probability that ant *k* transfers from city *i* to city *j* is:

(3) $$p_{ij}^{k}(t)=\left\{\begin{array}{c} \frac{{\left[\tau_{ij}(t)\right]}^{\alpha }\cdot {\left[\cap_{ij}(t)\right]}^{\beta }}{\sum_{s\in J_{k}(i)}{\left[\tau_{is}(t)\right]}^{\alpha }\cdot {\left[\cap_{is}\right]}^{\beta }},\;\;\;\;\;\;while\;j\in J_{k}\left(i\right)\\ 0,\;\;\;\;\;\;\;\;\;\;\;\;\;\;\;\;\;\;\;\;\;\;\;Others \end{array}\right.$$

where $J_{k}\left(i\right)=\left\{1,2,3\ldots n\right\}-{tabu}_{k}$ represents the set of cities that ant k is allowed to choose in the next step. Among them, the taboo list(*tabu_k_*) records the cities that ant k has passed through so far. When all *n* cities are added to the taboo list(*tabu_k_*), ant k has completed a tour. At this time, the path that ant *k* has traveled is a feasible solution to the traveling salesman problem (TSP), where $\cap_{ij}$ is a heuristic factor, representing the expected degree of an ant’s transfer from city *i* to *j*. In the ACO, it is usually taken as the inverse of the distance between city *i* and *j*. *α* and *β,* respectively, represent the relative importance of pheromones and the expected heuristic factor. And when all the ants complete one tour, the pheromone on each path is updated according to the following formula:

(4) $$\tau_{ij}(t+n)=(1-\rho )\cdot \tau_{ij}\left(t\right)+{∆\tau }_{ij}$$

where ρ (0 < ρ < 1) and 1 − *ρ* represent the evaporation coefficient of pheromone on the path and the persistence coefficient of pheromone, respectively. ${∆\tau }_{ij}\;$represents the increment of pheromone on edge *ij* in this iteration, that is:

(5) $${∆\tau }_{ij}=\sum_{k=1}^{m}∆\tau_{ij}^{k}$$

Among them, $∆\tau_{ij}^{k}$ represents the amount of pheromone left on edge *i* and *j* by the *k*th ant in this iteration. If ant *k* does not pass through edge *i* and *j*, then $∆\tau_{ij}^{k}$ = 0. It can be expressed as:

(6) $$∆\tau_{ij}^{k}=\left\{\begin{array}{c} \frac{Q}{L_{k}},\;\mathrm{When ants pass through edge ij during this round trip}\\ 0,\;\;\;\;\;\;\;\;\;\;\;\;\;\;\;\;\;\;\;\;\;\;\;\;\;\;\;\;\;\;\;\;\;\;\;\;\;\;\;\;\;\;\;\;\;\;\;\;\;\;\;\;\;\;\;\;\;\;\;\;\;\;\;\;\;\;Others \end{array}\right.$$

where *Q* is a normal number, *L_k_* represents the length of the path traveled by the *k*th ant in this tour.

### 2.4. Stacking Ensemble Learning

To some extent, each learning model has some limitations and cannot fully adapt to the data, nor can the accuracy reach 100%. In order to further improve the accuracy of prediction, it is possible to consider combining multiple models to fully utilize the advantages of each model, from which the best model can be selected. This method is also known as an ensemble learning algorithm. Ensemble learning algorithms can be classified into bagging, boosting, and stacking ensemble learning based on their types [56]. While the bagging and boosting algorithms use the principles of voting and weighted averaging, respectively, the stacking algorithm takes a different approach by constructing a new model to retrain the predictions of multiple learners. The basic idea is to use multiple base learners to learn from the training data separately and then use the outputs of these base learners as new features to input into the meta-learner for training. Finally, the meta-learner provides the prediction results.

Specifically, stacking ensemble learning consists of two stages [57]. In the first stage, different base learners are used to train the original training data to obtain a variety of very different predictions. Among them, these base learners can be different types of algorithms, such as decision trees, support vector machines, neural networks, etc. In the second stage, the prediction results of the base learners in the first stage are utilized as new features and combined with the labels of the original training data to form a new training set for training meta-learners. Among them, the meta-learner is usually a comparatively simple model, such as linear regression, logistic regression, and so on. In general, it can be said that in the testing stage, the base learners are firstly used to predict the new test data. And then these prediction results are input into the meta-learner to obtain the final prediction result.

It is necessary to discuss in detail its two stages. Suppose we have a training set containing *N* samples *D* = {(*x*_1_, *y*_1_), (*x*_2_, *y*_2_), …, (*x_N_*, *y_N_*)}, where *x_i_* and *y_i_* are the input feature vector and the corresponding label, respectively. We have *M* base learners *L*_1_, *L*_2_, …, *L_M_* and one meta-learner *L*_meta_.

➀ The first stage. Each base learner $L_{m}$ (*m* = 1, 2, …, *M*) is trained on the training set D to obtain the model $L_{m}$(*D*). Where, different base learners can be based on different algorithms. For example, model *L*_1_ can be obtained by training on decision tree algorithm and model *L*_2_ can be obtained by training on support vector machine algorithm.

For each sample $x_{i}$, a prediction is made using the base learner to obtain the prediction result $z_{i,m}=L_{m}$($x_{i}$), where *m* = 1, 2, …, *M*. For example, for a binary classification problem, the base learner might output the probability value that the sample belongs to a certain category, while for a regression problem it outputs the specific predicted value.

➁ The second stage. Construct a new training set $D^{\prime }=D=\left\{\left(z_{1},y_{1}),(z_{2},y_{2}),\ldots ,(z_{N},y_{N}\right)\right\}$, where $z_{i}=(z_{i,1}$,$z_{i,2}$, …, $z_{i,M}$). In this step, the prediction results of each base learner for each sample obtained are combined to form a new feature vector that contains the combined learning information of the different base learners for the sample.

Utilize the new training set $D^{\prime }$ to train the meta-learner $L_{meta}$ to obtain the model $L_{meta}$($D^{\prime }$). In this case, the meta-learner can be a simpler linear model (e.g., linear regression, logistic regression) or a more complex one, such as a nonlinear model. When training the meta-learner, we need to consider the algorithm and loss function it chooses, e.g., in regression problems, there is a mean square error loss function; in classification problems, there is a cross-entropy loss function, etc. The parameters of the meta-learner are tuned by the gradient descent method. This tuning allows the meta-learner to learn how to optimally combine the predictions of each base learner.

## 3. Experiment and Results

### 3.1. Evaluation Metrics

**Figure 3:**
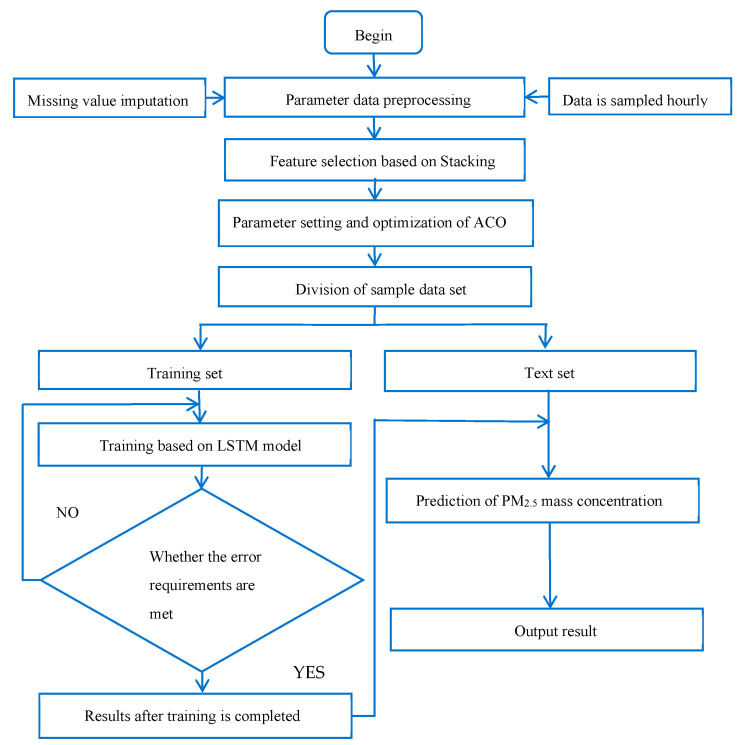
Flowchart of the model.

The flowchart of optimizing the LSTM prediction model by ACO and the stacking ensemble learning method (see Figure 3). The main purpose of this is to further render the initial values and thresholds of the neural network more reasonable, thus improving the convergence speed of the neural network and finding the optimal solution. In this model, the LSTM neural network mainly serves as a predictor. In the ACO optimization part, the optimized ACO is mainly employed. This algorithm is utilized to set and optimize the parameters of the overall model. Finally, the obtained optimal weight and threshold are applied to the LSTM neural network. The main role of the stacking ensemble learning method is to perform feature selection on the overall model. The specific implementation steps are as follows:

### 3.2. Parameter Settings

When constructing the stacking-ACO-LSTM model, the key parameters were carefully set and tuned (Table 1).

Among them, the value range of the number of ants is set as [10, 20, 30, 40, 50]. This parameter determines the number of ants participating in the search in each iteration. The more the number of ants, the more hyper parameter combinations the algorithm can explore in each iteration. However, it will also increase the computational load at the same time.

The number of neurons in the hidden layer of the LSTM is set with several options: [16, 32, 64, 128]. Different numbers of neurons will significantly affect the model’s ability to learn and represent data features. If the number is too small, the model may not be able to fully capture the complex patterns in the data. On the other hand, if the number is too large, it may lead to over-fitting and a waste of computational resources.

As an important means to prevent over-fitting, the value range of dropout is set as [0.2, 0.3, 0.4, 0.5]. Dropout randomly discards some neuron connections during the training process, reducing the co-adaptation degree among neurons and enhancing the generalization ability of the model. An appropriate dropout value can effectively balance the training effect of the model and the risk of over-fitting.

The value range of the number of epochs for model training is [20, 30, 40, 50, 60]. The number of training epochs determines how many times the model learns from the training data. If the number of epochs is too small, the model may not converge sufficiently and fail to learn the inherent patterns in the data. Conversely, if the number of epochs is too large, over-fitting may occur, resulting in the model’s reduced adaptability to new data.

Through the optimization of the stacking ensemble learning method and the ant colony algorithm, the number of ants finally selected in the model is 30, and this quantity shows the best accuracy in prediction. The number of neurons in the LSTM hidden layer is selected to be 32, which performs excellently in balancing model complexity and learning ability. A dropout value of 0.2 is chosen, which effectively prevents over-fitting while maximizing the model’s learning ability. The number of epochs is set to 50, enabling the model to fully converge on the training data and avoiding over-fitting. As a result, the stacking-ACO-LSTM model achieves relatively ideal performance.

### 3.3. Prediction Methods

This paper selects the sliding window prediction method. Its core idea is to slide a fixed-size window over the data sequence, moving one time step or one data point each time. In this way, the original data are transformed into multiple subsequences, which can be used for training the model or making predictions. This prediction method can effectively capture the dynamic changes of the time series.

### 3.4. Results

**Figure 4:**
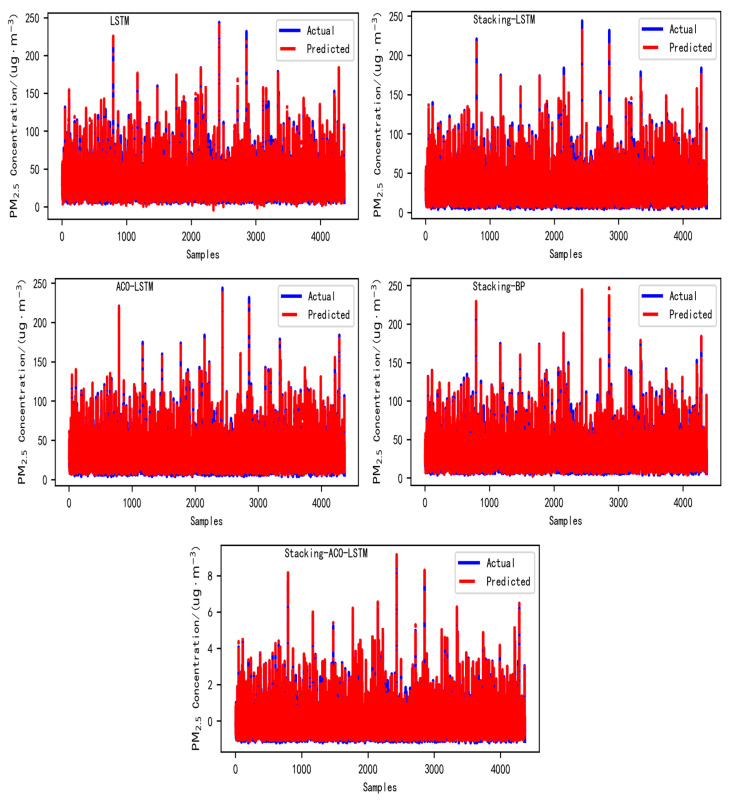
Prediction curves of five models.

In this experiment, five different experiments were mainly carried out, namely LSTM, LSTM based on the stacking ensemble learning method (stacking-LSTM), LSTM based on ACO (ACO-LSTM), BP neural network based on the stacking ensemble learning method (stacking-BP), and LSTM based on the stacking ensemble learning method with ACO (stacking-ACO-LSTM). In this way, the gap between the real value and the predicted value can be seen more clearly. The predicted curve is shown in Figure 4. In the experiment, a total of three evaluation indicators, namely regression coefficient of determination (R^2^), mean square error (MSE), and mean absolute error (MAE), are employed to evaluate the five models. The evaluation results are shown in Table 2 below.

As shown in Table 2 below, among all the tested models in Nanchang City, they include the LSTM network optimized by stacking ensemble learning, the BP neural network optimized by stacking ensemble learning, and the LSTM network optimized by the ACO algorithm. From the data in the table, it can be seen that the performance of these three optimization methods is at a moderate level. Their mean squared error (MSE), mean absolute error (MAE), and coefficient of determination (R^2^) are 36.516, 4.480, and 0.939; 36.085, 4.154, and 0.939; and 36.602, 4.590, and 0.938, respectively. Compared with the unoptimized LSTM model, the improvement in performance is not prominent. In view of this, on the basis of optimizing the LSTM network using stacking ensemble learning, the ACO algorithm is introduced to jointly optimize it, thus constructing the stacking-ACO-LSTM model. The mean squared error (MSE), mean absolute error (MAE), and coefficient of determination (R^2^) of this model are 0.058, 0.178, and 0.942, respectively. Compared with the model using only the LSTM network, the mean squared error (MSE) and mean absolute error (MAE) have decreased by 99.88% and 96.58%, respectively, and the coefficient of determination (R^2^) has increased by 2.39%. From the results of the mean squared error (MSE) and mean absolute error (MAE), it can be clearly found that the performance of the stacking-ACO-LSTM model surpasses that of other models. However, the improvement in its coefficient of determination (R^2^) compared with other models is not significant. To further analyze the advantages and disadvantages of these models and evaluate their generalization ability, the data of two other prefecture-level cities in Jiangxi Province, namely Ganzhou City and Jiujiang City, are introduced, and this is achieved by comparing the prediction performance of several models on the data of Nanchang City, Ganzhou City, and Jiujiang City. From the integrated table data, it can be seen that, regardless of whether it is Ganzhou City or Jiujiang City, the optimization effects of the final optimized stacking-ACO-LSTM model in terms of mean squared error (MSE) and mean absolute error (MAE) far exceed those of other models, and its coefficient of determination (R^2^) is also slightly higher than that of other models. Nevertheless, it is found from Table 2 that the correlation results of Jiujiang City are worse than those of the other two cities. We believe that the reason why the correlation results of Jiujiang City are not as ideal as those of Nanchang City and Ganzhou City mainly lies in the significant fluctuations in the particulate matter data of Jiujiang City. In addition, environmental factors such as weather conditions and air humidity may also have an impact on this correlation. It should be noted that the core of this paper focuses on the predictive ability of the optimized model. Although the correlation index of Jiujiang City is relatively low, its predictive accuracy within its own optimized model is still at a good level. Thus, this result still has academic value and analytical significance and can effectively support the discussion on the effectiveness of the model in this paper. Conclusively, by comprehensively considering the prediction situation of Nanchang City and taking into account the generalization ability of the model, the final stacking-ACO-LSTM model demonstrates the best prediction ability.

**Table 2:** Model evaluation results of three cities.

City	Model	MSE	MAE	R^2^
Nanchang	LSTM	47.586	5.206	0.920
	Stacking-LSTM	36.516	4.480	0.939
	ACO-LSTM	36.602	4.590	0.938
	Stacking-BP	36.085	4.154	0.939
	Stacking-ACO-LSTM	0.058	0.178	0.942
Ganzhou	LSTM	49.411	4.976	0.894
	Stacking-LSTM	45.176	4.790	0.904
	ACO-LSTM	41.869	4.659	0.911
	Stacking-BP	46.058	4.881	0.902
	Stacking-ACO-LSTM	0.089	0.215	0.920
Jiujiang	LSTM	204.332	10.221	0.709
	Stacking-LSTM	189.219	9.864	0731
	ACO-LSTM	193.934	9.804	0.724
	Stacking-BP	196.889	9.845	0.720
	Stacking-ACO-LSTM	0.248	0.357	0.741

**Figure 5:**
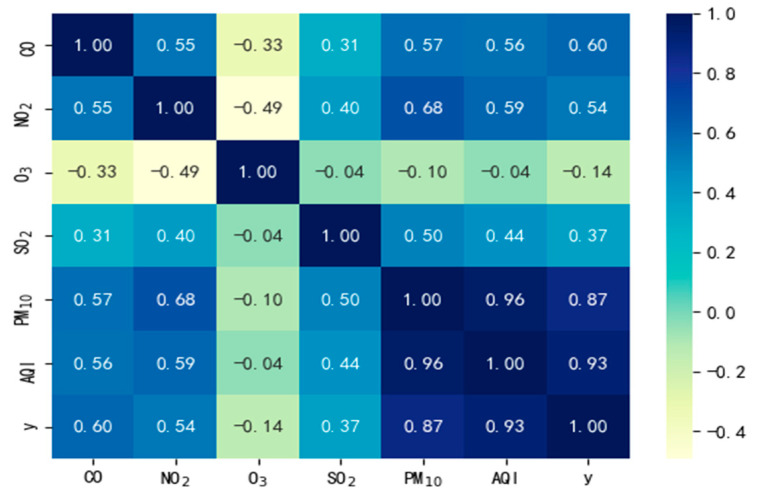
Correlation heatmap analysis.

In this paper, on the basis of previous research results, five relevant pollutant indicators affecting PM_2.5_ in Nanchang were selected, and correlation analyses were conducted before modeling. The analysis results are shown in Figure 5, where PM_2.5_ concentration is represented by *y*. It is not difficult to see from Figure 5 that the correlation between AQI, PM_10_, and PM_2.5_ is extremely significant and highly close. While the correlation between CO, NO_2_, SO_2_, and PM_2.5_ is moderate. The correlation between O_3_ and PM_2.5_ concentration is the lowest. Among these substances, AQI is relatively special. It is an index calculated by comprehensively considering the concentrations of various air pollutants. Therefore, treating these particulate matters is equivalent to treating AQI itself. In conclusion, when fully addressing PM_2.5_ particulates in haze control, the focus of treatment should first be on PM_10_. This is because the high correlation between PM_10_ and PM_2.5_ means that effective treatment of PM10 will greatly facilitate progress in PM_2.5_ treatment. Secondly, the management of CO, NO_2_, and SO_2_ should not be overlooked. Although their correlation with PM_2.5_ is not as close as that of PM_10_, they still have an important impact on air quality and should be given sufficient attention in the treatment process. In this system, O_3_ has an extremely low correlation with PM_2.5_ concentration and is regarded as a factor that can almost be ignored. However, this does not mean that O_3_ is completely ignored. In the overall air quality treatment plan, we need to consider the actual situation. Under the premise of ensuring the realization of the main goals, O_3_ can be given a certain degree of attention and management.

## 4. Conclusions

In the process of using the ant colony optimization to optimize the parameters of the model, the selection of the number of ants and the maximum number of iterations when the algorithm runs is very important. Their values will be crucial for the whole model. In the optimal model stacking-ACO-LSTM, the number of ants chosen at the beginning of this paper is 20, and the maximum number of iterations is 2. At this time, the model effect can reach 0.940. However, this is probably not the optimal result. So it is considered to gradually increase the number of ants and the maximum number of iterations. When the number of ants is 30 and the maximum number of iterations is 3, the model effect reaches its optimal state, which can reach 0.942. As both the number of ants and the maximum number of iterations increase upwards, the results become worse. Therefore, the model reaches its optimum when the number of ants is 30 and the maximum number of iterations is 3.

The stacking-ACO-LSTM model performs exceptionally in the prediction of PM_2.5_ concentration in Nanchang City. Among them, the introduced deep learning LSTM framework plays a crucial role. It utilizes its own advantages in processing time-series data to overcome the problems of unstable samples, long running time, and unstable and inaccurate results of traditional prediction methods. Meanwhile, the combination of the stacked ensemble learning method and the ant colony optimization method further optimizes the model parameters and improves the prediction performance of the model. The predicted values of the combined model are closest to the real values compared with other models, which is highly practical.

Accurate prediction results of PM_2.5_ content can provide a data basis for air quality prediction. At the same time, some measures can be taken in advance to solve the air pollution problem based on the results obtained, which can effectively improve urban air quality and provide some suggestions for green traveling. In the process of the experiment, the initial model lacked parameter optimization measures for the overall model. Consequently, the obtained result might not be the optimal one. In view of this, the ant colony optimization method is used in this paper to find the optimal hyperparameters of the integration model. There are numerous and complex particulate substances in the air, which have nonlinear characteristics. In this paper, the stacking ensemble learning method is used to perform weight analysis on the feature variables, that is, the factors that can affect PM_2.5_. By filtering out the feature variables with larger weights, the computational amount of the model prediction is reduced, and the accuracy of the prediction model is improved. Through the experimental comparison and analysis, it can be seen that the stacking-ACO-LSTM model proposed in this paper has the highest prediction accuracy of PM_2.5_ concentration among these models.

## Figures and Tables

**Table 1:** Parameter search space.

Parameter	Range of Variation
The number of ant	[10, 20, 30, 40, 50]
The number of neurons of the LSTM	[16, 32, 64, 128]
Dropout	[0.2, 0.3, 0.4, 0.5]
The number of epochs in model training	[20, 30, 40, 50, 60]

## Data Availability

Sequence data and source code that support the findings of this study have been deposited online at https://doi.org/10.5281/zenodo.15151495.
